# The Role of Emotional Valence for the Processing of Facial and Verbal Stimuli—Positivity or Negativity Bias?

**DOI:** 10.3389/fpsyg.2019.01654

**Published:** 2019-07-26

**Authors:** Christina Kauschke, Daniela Bahn, Michael Vesker, Gudrun Schwarzer

**Affiliations:** ^1^Department of German Linguistics, Philipps-University Marburg, Marburg, Germany; ^2^Department of Developmental Psychology, Justus-Liebig-University Gießen, Gießen, Germany

**Keywords:** emotion, valence, word processing, face processing, positivity bias, negativity bias, development

## Abstract

Emotional valence is predominately conveyed in social interactions by words and facial expressions. The existence of broad biases which favor more efficient processing of positive or negative emotions is still a controversial matter. While so far this question has been investigated separately for each modality, in this narrative review of the literature we focus on valence effects in processing both words and facial expressions. In order to identify the factors underlying positivity and negativity effects, and to uncover whether these effects depend on modality and age, we present and analyze three representative overviews of the literature concerning valence effects in word processing, face processing, and combinations of word and face processing. Our analysis of word processing studies points to a positivity bias or a balanced processing of positive and negative words, whereas the analysis of face processing studies showed the existence of separate positivity and negativity biases depending on the experimental paradigm. The mixed results seem to be a product of the different methods and types of stimuli being used. Interestingly, we found that children exhibit a clear positivity advantage for both word and face processing, indicating similar processing biases in both modalities. Over the course of development, the initial positivity advantage gradually disappears, and in some face processing studies even reverses into a negativity bias. We therefore conclude that there is a need for future research that systematically analyses the impact of age and modality on the emergence of these valence effects. Finally, we discuss possible explanations for the presence of the early positivity advantage and its subsequent decrease.

## Introduction

### Emotional Valence in Human Perception and Processing

Emotional stimuli are highly relevant for human behavior since humans have to process such stimuli very quickly in order to detect and react to events important for our survival. General effects of emotionality i.e., a processing advantage for emotional as compared to neutral information, have been shown for various types of stimuli, including words (e.g., Kanske and Kotz, 2007; Kousta et al., 2009; Yap and Seow, 2013; Goh et al., 2016) and faces (Johansson et al., 2004; Groß and Schwarzer, 2010). In general, the emotional significance of a stimulus enhances its processing (Zeelenberg et al., 2006).

One very basic feature of emotional stimuli is their hedonic valence. Valence refers to the pleasantness or unpleasantness of an emotional stimulus. Nearly all events and experiences, such as faces, sounds, music, art, pictures, written or spoken language, and many others can be classified along this dimension as more or less positive or negative. Given that valence is such a crucial factor for the representation and categorization of human experience, efforts have been made to investigate whether the proximity of stimuli toward either end of this dimension's continuum (positive or negative) leads to preferential processing, and if so, which one. In the literature, a marked difference in responding to negative or positive stimuli has been labeled as “bias.” Although there is empirical evidence for existing asymmetries in the way humans use positive and negative information (Vaish et al., 2008), the precise direction of such valence biases is still unclear. In cases when either positive or negative stimuli are perceived and processed differentially, the response to the preferred category is described with terms like “advantage,” “preference,” “superiority,” “enhancement,” or “facilitation,” while the other category, accordingly, shows a “disadvantage” or “delay.” Evidence for a bias is usually associated with a behavioral advantage or facilitation in experimental tasks, in particular with higher accuracy scores or faster response times in the context of stimuli with a specific valence. Thus, “bias” can be defined as a behavioral advantage reflected by better or faster reactions toward positive or negative stimuli. In this regard, empirical findings are heterogeneous: Apart from studies that demonstrated a behavioral preference for either positive or negative stimuli, other studies found no asymmetries. These inconsistent results may be due to methodological factors such as age, stimulus modality, or task. For example, Bayer and Schacht (2014) found that adults process positive words faster than negative ones, but show the reverse pattern for emotional pictures and facial expressions. Therefore, the first and main aim of the present paper is to review the literature for behavioral valence effects taking age, modality, and task into consideration. Beyond the systematic compilation of valence effects in the literature, the question arises how and why behavioral biases emerge and what kind of underlying mechanisms may be responsible for them.

### Positivity and Negativity Biases

According to the notion of the “positivity bias,” there is a processing advantage of positive over negative stimuli. Positivity effects have been shown across modalities. Studies using verbal stimuli revealed evidence for a positivity advantage in various tasks with adults. For example, Bayer and Schacht (2014), Goh et al. (2016), and Hofmann et al. (2009) showed that positive words generally elicit faster reaction times than negative words in lexical decision tasks, with similar effects being found in semantic (Goh et al., 2016) and emotional categorization (Feyereisen et al., 1986) tasks. Sylvester et al. (2016) confirmed an advantage for positive words for children between 9 and 12 years of age who participated in an emotional categorization task. A positivity bias has also been found with regard to faces. For example, Feyereisen et al. (1986) and Leppänen and Hietanen (2004) demonstrated a speed advantage for positive stimuli in the decision whether a face is positive, negative or neutral. Similarly, Walden and Field (1982) showed that adults and children recognize happy faces with the fewest errors.

Several potential explanations have been offered for positivity superiority effects: One of the offered explanations is that negative emotions in general are less clustered and more distinct from one another than positive emotions, and are therefore more difficult to identify without confusion (Leppänen and Hietanen, 2004; Nummenmaa and Calvo, 2015). For language specifically, the informational density hypothesis claims that positive verbal stimuli are better elaborated and interconnected in memory than negative material (Unkelbach et al., 2008; Sylvester et al., 2016). For faces, two additional possible explanations for the positivity bias have been proposed, including that positive facial expressions may be more visually distinct than negative expressions, resulting in a visual saliency advantage, and that positive expressions might be differently encoded and processed compared to negative expressions (Leppänen and Hietanen, 2004; Nummenmaa and Calvo, 2015).

A “negativity bias” refers to the opposite pattern, a preferential processing of negative information. Evidence for this effect can also be found for verbal and non-verbal stimuli. For example, Nasrallah and Carmel (2009) demonstrated enhanced sensitivity to negative word valence in a categorization task with adults, and suggest that negative stimuli enjoy preferential access to perceptual processing. Similarly, Dijksterhuis and Aarts (2003) showed a preferential detection of subliminally presented negative word stimuli. For faces, a negativity bias has also been shown in multiple studies, particularly those which required participants to detect target faces amongst distractor stimuli. For instance, angry faces seem to be often easier to detect for adult participants amongst distractors than happy faces (Fox et al., 2000; Ohman et al., 2001; Fox and Damjanovic, 2006; Horstmann and Bauland, 2006; Pinkham et al., 2010). A negativity bias has also been proposed for child development: Vaish et al. (2008, p. 383) state that “infants attend more to, are more influenced by, and use to a greater degree negative rather than positive facets of their environment.” Indications of the existence of a negativity bias in infancy include, according to Vaish et al., a stronger orientation toward fearful than toward happy faces and a more elaborated verbal discourse about negative experiences than about positive experiences.

Explanatory accounts for the negativity bias emphasize critical evolutionarily adaptive functions, since it is important for survival to quickly detect, attend to and avoid negative, aversive stimuli (Baumeister et al., 2001; Vaish et al., 2008). However, it has been pointed out (Estes and Adelman, 2008a,b; Estes and Verges, 2008) that this “automatic vigilance” for negative stimuli might also bring about a behavioral disadvantage. On the one hand, humans pay more attention to and give more weight to negative than positive experiences. On the other hand, aversion of threatening stimuli may be more time-sensitive than attainment of appetitive stimuli. A possible consequence is that negative stimuli may elicit relatively slow responses on cognitive tasks. The authors refer to this generally prolonged responses to negative stimuli as a “negative delay.” If the particular relevance of negative stimuli leads to delayed disengagement of attention, longer reaction times may be required for the processing of negative compared to positive stimuli. In this sense, behavioral positivity effects may also result from a prolonged disengagement from negative stimuli.

### Developmental Changes in Valence Effects

Valence effects not only vary across tasks and modalities, but also change with age. Developmental changes have been observed in word processing. Although Russell and Ridgeway (1983, p. 802) showed that “children seem to organize emotion terms in much the same way as do adults” across the dimensions of valence and arousal, Widen and Russell (2008) concluded that emotion categories develop gradually, having investigated the comprehension, categorization, and production of emotion terms in preschoolers. Kauschke et al. (2012) showed improvements in the processing of abstract terms (including emotion words) using a lexical decision task with children between 8 and 12 years of age. Baron-Cohen et al. (2010) reported substantial changes in the size of the receptive emotion vocabulary between 4 and 11 years of age. These findings suggest that there are changes in the way children process words with emotional content. However, these studies do not allow for any conclusions about potential developmental changes with respect to valence effects.

Clear developmental changes have also been shown for the processing of facial stimuli: While infants younger than 6 months prefer positive facial expressions, they begin to pay more attention to negative expressions later in the first year of life (Ludemann and Nelson, 1988). Three-year old children recognize positive faces better than negative ones, but this difference seems to be weaker in older children (Freitag and Schwarzer, 2011). Gao et al. (2010), studying children between 7 and 14 years of age, showed that reactions of older children are more similar to those of adults than those of younger children, and concluded that an adult-like representation of facial expressions develops gradually during childhood.

### Aims and Questions

Overall, it seems well-documented that emotional valence influences human behavior and cognition. So far, it is still unclear whether it is mainly positive or negative information that facilitates processing, how valence effects are modified by stimulus modality, task and age, and what underlying mechanisms are responsible for apparent behavioral biases. The present paper thus seeks to shed light on the role of emotional valence for the processing of emotional stimuli.

In a narrative review of the literature, we focus on two types of stimuli that are highly important for human communication of emotions: Words and faces. In face-to-face interactions, emotions are usually communicated simultaneously by verbal and facial cues. During childhood, face-to-face communication between children and their adult caretakers is the primary opportunity for children to extract the meaning of words. Thus, facial information usually accompanies verbal information. Nevertheless, previous research mostly investigated the processing of emotional information of words and faces separately. Due to the high degree of ecological co-occurrence of emotional information from speech and faces, our aim was to look at the parallels and differences to be found among the results of studies on these two sensory modalities.

Therefore, the key questions guiding our review are as follows:

1. Do humans tend to prefer positive or negative stimuli when they perceive verbal and/or facial information?
2. Are potential valence preferences modality-specific, i.e., do we find parallels or differences with respect to valence effects in the processing of words and faces?
3. Do valence effects change with age, and if so, do similar developmental changes occur in both modalities?

To answer these questions, we present three representative reviews of the literature concerning valence effects in the processing of emotional stimuli. First, we consider studies on word processing in adults (Supplementary Table 1A) and children (Supplementary Table 1B), followed by a review on valence effects in face processing in children and adults separated by the methods used (Supplementary Table 2A, detection-based studies, Supplementary Table 2B, identification-based studies). Finally, we present a third review concerning valence effects in studies that use words as well as faces as stimuli (Table 1). The exact selection criteria of each review are explained below. We focused on publications that report empirical, behavioral results concerning valence effects, i.e., an explicit comparison of positive and negative stimuli was required. In line with the majority of the literature, a “bias” or processing advantage was assumed in case of significantly higher accuracy or faster response times for stimuli of a specific valence category.

**Table 1:** Studies on valence effects with faces and words as stimuli.

**Paper number**	**References**	**Participants**	**Method/Task**	**Stimuli**	**Results**	**Valence effect direction**
1	Bahn et al., 2017; Vesker et al., 2018a	n in total = 120 5-, 6-, 9-, 12-year-olds and adults, each group: *n =* 24 (12 female), native German speakers	Emotional categorization: “positive vs. negative”	German; 48 audibly presented emotion words: 24positive, 24 negative All words were recorded with a non-emotional neutral tone	- More correct reactions for positive compared to negative words in 5- and 6-year-olds and adults - No effect of valence in accuracy in 9- and 12-year-olds - Faster reactions for positive compared to negative words in 5- and 6-year-olds - No effect of valence in reaction times in 9- and 12-year-olds and adults	ACC Words: Adv-pos in 5- and 6- year-olds and adults Pos = neg in 9- and 12- year-olds RT Words: Adv-pos in 5- and 6-year-olds Pos = neg in 9- and 12-year-olds and adults
		n in total = 96 6-, 9-, 12-year-olds and adults, each group: *n =* 24 (12 female), native German speakers		24 positive, 24 negative emotional faces	- More accurate responses to positive faces in 6-year-olds, no significant differences in 9- and 12-year olds, and more accurate responses to negative faces in adults - Faster correct responses for positive faces for 6-, 9-, and 12-year olds, with no differences found in adults	ACC Faces: 6, 9, 12 year olds: Adv-pos Adults: Pos = neg RT Faces: 6 year olds: Adv-pos 9, 12 year olds: Pos = neg Adults: Adv-neg
2	Feyereisen et al., 1986	*n =* 16 (all female), age = 20-25 years, native French speakers	Emotion categorization: “happy” vs. “sad”	Written words: 8 positive, 8 negative emotion words, 8 positive, 8 negative faces	- Positive words faster than negative words - No RT difference between positive and negative faces	RT Words: Adv-pos RT Faces: Pos = neg
			same/different-judgement		- Positive faces faster than negative faces - Smaller effect for words	RT: Words: Adv-pos RT: Faces: Adv-pos
3	Rellecke et al., 2011	*n =* 24 (11 female), 18-35 years, native German speakers	EEG, Face-Word- Discrimination (determining whether each stimulus is a face or a word)	150 faces: angry - happy - neutral 150 written words: positive - negative - neutral	- Words faster than faces - No influence of valence	RT Words: Pos = neg RT Faces: Pos = neg
4	Schacht and Sommer, 2009	*n =* 24 (16 female), mean age = 23.5 years, native German speakers	EEG, Lexical Decision	Written words 120 verbs, positive - negative - neutral 120 non-words	- Negative and positive words faster than neutral - Accuracy: no difference between negative, positive and neutral words	RT Words: Pos = neg Acc Words: Pos = neg
			EEG, Face Decision Task (determining whether each presented face was normal, or partially smeared/blurred)	240 Faces: Angry, happy and neutral	- Happy and neutral faces faster than angry faces - Accuracy: No difference between negative, positive and neutral faces	RT Faces: Adv- pos Acc Faces: Pos = neg

*ACC, Accuracy; RT, Reaction Times; Adv-pos/neg, behavioral advantage for stimuli with positive/negative valence; pos = neg, no significant difference between positive and negative stimuli. Missing details about the participants' age and gender breakdown (see column “participants”) are due to missing information in the relevant publication*.

## Review 1: Valence Effects in Word Processing

In order to get an overview of the current state of research, we first checked reference lists in relevant papers and consulted computerized databases (Google scholar, Pubmed, Psycinfo, Psyclit, Medline, Msyndex), using the search terms “valence,” “emotion,” “word processing,” and different combinations of these terms. Studies were included if they met the following criteria:

- Studies must report behavioral results on uni-modal word processing. Cross-modal priming tasks with words as targets were excluded in order to avoid influences of stimuli from other modalities (e.g., faces or emotional pictures) on word processing.
- The selected studies cover different behavioral tasks: lexical decision, categorization (positive/negative, emotional/neutral, concrete/abstract), word identification tasks, word counting tasks, emotional stroop tasks, translation tasks, reading tasks, or memory tasks.
- If studies used psycho-physiological measures such as EEG, fMRT, or pupillary responses, these studies were also included, but only the behavioral results were extracted for the review.
- Target stimuli used in the studies had to be words differing with respect to valence (positive/negative). Positive and negative words had to be compared directly in a statistical analysis.
- Reported outcomes had to be accuracy and/or reaction times.
- Participants had to be healthy children or adults.

### Search Results for Adult Studies

**Figure 1:**
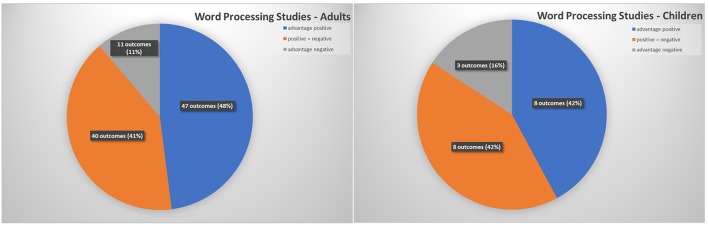
Distribution of outcome measures in word processing studies (absolute numbers based on 46 publications including 98 outcome measures for adults and 9 studies including 19 outcome measures for children).

Based on these criteria, Supplementary Table 1A presents a comprehensive overview of the findings from 42 relevant publications reporting original research on word processing with healthy adult participants. Another four studies, listed in Table 1, investigated word combined with face processing. The 46 publications report 98 outcome measures. Figure 1 illustrates the distribution of all word-related outcome measures reported in the studies (for adults: based on Supplementary Table 1A, Table 1). The general picture that emerges is quite clear: A minority of studies (11%) found better or faster processing of negative words. Studies that revealed better and/or faster processing of words with a positive valence or did not find differences between positive and negative word stimuli clearly predominate. In other words, there is only weak evidence for an advantage of negative over positive stimuli in adults' word processing. Typically, positive and negative words are either processed with the same speed and/or accuracy, or positive words enjoy a processing advantage.

### Search Results for Studies With Children

In addition to the literature search describe above, we searched specifically for studies that investigated valence effects in word processing with typically-developing children as participants, using the search term combinations “positive OR negative OR neutral AND word^*^ AND children,” “emotion^*^ AND word^*^ AND children,” and “valence AND word^*^ AND children.” After checking several hundred titles and abstracts, nine publications remained that explicitly address valence effects in children's or adolescents' word processing according to our criteria (see Supplementary Table 1B, Table 1 for details). Compared to a relatively large body of research with adult participants, there seems to be a striking lack of empirical findings for children, but quantity of research in this field has expanded in recent years. The age of the participants in these studies ranged from 5 to 17 years. Only three of 19 outcome measures (16%) revealed an advantage for negative words. In all other cases, either an advantage for positive words or a balanced processing of positive and negative words was found (see Figure 1, for children: based on Supplementary Table 1B, Table 1). The existing studies with child participants thus converge with the adult data, in that they do not support a negativity advantage in word processing, but rather point to a tendency toward a positivity bias, at least in particular age groups.

Five studies that included different age groups shed light on age-related changes during childhood. In a cross-sectional study with five- six-, nine-, and 12-year olds (Bahn et al., 2017, see Supplementary Table 1B, Table 1), 5- and 6-years old children showed an early behavioral advantage for positive over negative words in two word processing tasks (lexical decision and emotional categorization), with a gradual diminishment of his effect in older children. Ponari et al. (2018) demonstrated a positivity advantage for accuracy in a lexical decision task, in particular in children aged 8–9 years, but not in younger and older children. Quas et al. (2016) compared children with a mean age of 8 and 13 years in a memory task. Among the 13-year-olds, accuracy was higher for negative than positive words, while in the younger group, accuracy was unrelated to word valence. Another study on word recall (Zhang et al., 2018) showed no age-related changes: Negative words were recalled better than positive words in two age groups (mean age 7.5 and 11.4 years, respectively).

### Evaluation

Although our overview revealed overall trends in favor of a positivity bias, some inconsistencies remain. Obviously, the results are modified by stimulus characteristics, task, outcome measures, and other factors. Some important methodological factors that reduce the comparability of the reviewed studies and influence the appearance, direction, or magnitude of valence effects in word processing are discussed below.

First, investigations of valence effects bear the risk of confounding valence with arousal. It has been widely debated how strongly valence effects in word processing are influenced by arousal (Larsen et al., 2006, 2008; Estes and Adelman, 2008a,b). Estes and Adelman (2008a) observed that negative words elicited slower lexical decisions and naming than positive words. However, negative words tend to have higher arousal values, i.e., are perceived with a higher intensity, than positive words. Thus, valence effects might be reduced to a higher arousal of negative stimuli. Larsen et al. (2008) reanalyzed the item set of Estes and Adelman (2008a) with a specific focus on arousal and found modulating effects: Among non-arousing stimuli positive words elicited faster lexical decisions than negative words, but among highly arousing stimuli this effect disappeared. When arousal was controlled for, longer reaction times for emotionally negative words were observed. Hofmann et al. (2009) also report a differential impact of arousal on the processing of negative and positive words: Whereas a behavioral facilitation to positive words (reflected by faster reaction times and fewer errors) was observed regardless of their arousal level, arousal seemed to modulate behavioral responses to negative words. The authors concluded that high arousal facilitates early processing of negative, but not positive words. Kousta et al. (2009) found a significant effect of processing emotional over neutral words even when arousal was held constant. In the study by Goh et al. (2016), positive words were processed faster than negative words, without an influence of arousal. Kuperman et al. (2014) concluded from their meta-study that negative valence as well as high arousal slow down word processing, that the effects of valence and arousal are independent, and that valence has a stronger effect on word processing than does arousal. Obviously, the possible interactions between valence and arousal are complex, and differences in arousal might explain the inconsistent findings of previous studies on word processing at least to some degree. In order to uncover valence effects, the arousal values of the positive and negative target items should be held constant.

A second relevant methodological factor with respect to stimulus selection in word processing studies relates to the semantics of the word stimuli. Most Studies use a large variety of very different word categories and parts-of-speech. For example, the Berlin Affective Word List (BAWL, Võ et al., 2009; Jacobs et al., 2015) contains ~3,000 German words. Among them are concrete words with relatively neutral meanings (e.g., “Ampel,” traffic light, or “Halle,” hall), abstract words with relatively neutral meanings (e.g., “Entwurf,” draft, or “Klassik,” classicism), concrete words with emotional connotations (e.g., “Spinne,” spider, or “Sonne,” sun), as well as emotion terms (e.g., “Abscheu,” disgust, or “Freude,” joy). Goh et al. (2016) selected only concrete words as target items for their tasks, while abstract terms were added as distractors in one of the tasks. Ponari et al. (2018) found that positive emotional valence facilitates the processing of abstract words, but not concrete words. They therefore conclude that emotional valence is especially useful for the acquisition of abstract words. Since, Yao et al. (2016) point out that the effects of valence and arousal on word processing are modulated by concreteness, caution is warranted when using a mixture of different (concrete and abstract) word categories. In most ratings of emotional valence, valence is attributed to concrete words that carry negative or positive connotations. Kuperman et al. (2014) name the words coffin, cotton, and kitten as examples of negative, neutral, and positive items. Connotations accompany almost all open word classes and can be defined as evaluative associations that reflect subjective, mostly idiosyncratic forms of appraising the referent (Klann-Delius, 2015). Of course, almost every word may be perceived as more or less positive or negative, depending on an individual's experience with the concept (e.g., the word cat may be evaluated as positive or negative depending on one having pleasant or frightening prior experiences with cats). In contrast to words with affective connotations, emotion terms (like anger or love) refer more directly to emotions as symbols. Emotion terms are items from the emotive vocabulary of the mental lexicon, and they encode internal states in a conventional linguistic envelope (Klann-Delius, 2015; Schwarz-Friesel, 2015). Importantly, valence is an inherent part of the semantic features that characterize emotion terms (e.g., joy refers to a positive emotion, irrespective of personal experience), while in the case of connotations valence comes as an additional, idiosyncratic, and culture-specific evaluation that accompanies the denotative meaning. Thus, the existence and direction of a valence effect may be modified by the selection of word stimuli. Vigliocco et al. (2013) underline the specific role of emotion terms in child development: internal affective experience provides at least an initial grounding to abstract concepts. Emotion terms are seen as precursors of successfully building an abstract vocabulary, since they serve as a “crucial stepping stone into the development of the ontological distinction between entities existing in the physical world and those existing only in the human mind” (Vigliocco et al., 2013, p. 2). Given that emotion terms are acquired earlier than other abstract words, and that evaluative connotations for concrete words are strongly age-related, emotion terms offer an appropriate semantic field for the study of valence effects in children.

Another stimulus property that might influence responses is the modality in which the words are presented. The majority of all studies used written words displayed on a computer screen, while only 7 of the 55 studies presented the words audibly via headphones. Of course, auditory presentation is especially relevant for young participants with limited reading skills. The pattern with respect to valence effects does not differ between the modalities of stimulus presentation: for written as well as for auditory stimuli, a negative advantage occurred less frequently than a positivity advantage or a lack of valence asymmetry. In case of auditory stimuli, the stimulus word may be presented in neutral or emotional tone. In six of the 7 studies, no information was given about the prosodic characteristics of the stimuli, and one study (Bahn et al., 2017) used verbal stimuli spoken in a neutral tone. Therefore, no conclusions can be confidently drawn about a possible influence of emotional vs. neutral tone.

The next relevant methodological aspect in word processing studies is task type. It can be assumed that the relevance of valence for responding to an affective stimulus is task-specific. The majority of the reviewed studies on word processing used one of three different task types: lexical decision tasks, memory tasks, or categorization tasks. For lexical decision, the participant has to decide whether a target stimulus (presented either visually or as a spoken auditory stimulus) is a word or a pseudo-word. This task emphasizes the word form and does not require full access to the word's meaning. Therefore, the valence of the word is less relevant and not necessary for the response. In memory tasks, a list of words is presented to the participants, after which they are asked to recall as many of the words from the list as they can remember or to indicate on a new list whether an item had been heard before or not. Emotional valence of the target word is also not directly relevant here, but the word's meaning, including information about its valence, is likely to be activated for recollection and retrieval. Another frequently used task type is semantic categorization, where participants have to assign word stimuli to distinct semantic categories. For example, the distinctions between positive and negative, between positive/negative and neutral, or between concrete and abstract have been used in categorization tasks. These tasks are more difficult as they require semantic analysis and direct access to the semantic properties of the words. In emotional categorization, valence has to be considered explicitly in order to categorize a word as positive or negative, making valence particularly response-relevant. Given the different cognitive requirements of the tasks and the differing role of emotional valence, the question arises whether specific tasks are associated with specific outcomes. Out of the 54 outcome measures on lexical decision reported in the tables, only 5% showed a negativity advantage, while 52% of the results were in favor of positive words, and the remaining 43% of the outcome measures did not show valence effects. Regarding memory tasks, the 16 outcome measures were distributed more equally: 38% showed a negativity advantage and 31% each a positivity advantage or no effect of valence. With respect to categorization tasks, 21% of the 24 outcome measures showed a negativity advantage, while 54% of the results were in favor of positive words and the remaining 25% showed no effect of valence. Obviously, the outcomes of lexical decision and categorization tasks reveal quite similar patterns (i.e., a tendency in favor of a positivity bias). In several studies that used lexical decision as well as categorization tasks with the same stimuli, no task-specific discrepancies emerged: Goh et al. (2016) showed that positive words were processed faster than negative words in lexical decision as well as in semantic categorization. In the study of Bahn et al. (2017) (see Supplementary Table 1B, Table 1), reactions also turned out to be task-independent: children aged 9 years or older as well as adults showed no behavioral differences between positive and negative words in lexical decision and in emotional categorization. In addition, a positivity advantage was evident for 5- and 6-years old children in both tasks. The results from lexical decision and emotional categorization obtained by Dijksterhuis and Aarts (2003) also showed converging results, with better reactions for negative words in both tasks. Only one study, however, found differential results for lexical decision and emotional categorization tasks. Estes and Verges (2008) observed slower reaction times for negative stimuli in lexical decision, but faster reactions in emotional categorization. The authors argue that response-relevance might explain their task-specific results. The observed slower reaction times for negative stimuli in lexical decision may reflect that a disengagement of attention is more difficult when negative stimuli have to be processed, whereas sustained attention to negative stimuli might have speeded up the response times in their categorization task. Compared to the results for lexical decision and categorization tasks, memory tasks stand out by their relatively high proportion of results in favor of negativity. In addition, out of the 14 findings across the three task types that were in favor of a negativity advantage, six (42%) were obtained by memory/recall tasks. To summarize, task type seems to influence responses in word processing (lexical decision and categorization tasks opposed to memory tasks), but there are no clear associations between the response-relevance of valence and the observed outcomes.

Finally, outcome measures have to be considered. Supplementary Tables 1A,B illustrate that the patterns of valence effects often do not converge for accuracy and reaction times, even when participants are facing the same stimuli. For example, when positive words are processed more accurately, this does not necessarily mean that the same set of words is also processed faster.

## Review 2: Studies on Valence Effects in Face Processing

To review the state of the literature regarding the existence of broad biases which favor more efficient processing and perception of positive or negative facial expressions, we conducted a search of existing studies in a similar manner as that previously described for the word literature review, including studies with adults and children: We searched Google Scholar, Pubmed, and Psycnet for relevant results using keywords such as “faces,” “emotion,” “positive,” and “negative.” Studies were included if they met the following general criteria:

- Studies must report behavioral results on uni-modal face processing. If studies used psycho-physiological measures such as EEG, fMRT, or pupillary responses, these studies were also included, but only the behavioral results were extracted for the review.
- Stimuli used in the studies had to be faces differing with respect to valence (positive/negative). Studies were included where positive and negative faces were compared directly in a statistical analysis in terms of behavioral results, or where a significant effect was present or absent for one category vs. the other.
- In papers with multiple experiments, the experiments were reported separately whenever they offered a direct comparison of positive vs. negative faces.
- Reported outcomes had to be accuracy and/or reaction times.
- Participants had to be healthy children or adults.

After checking several hundred study titles and abstracts for the purposes of our review we only included studies which provided behavioral measures to indicate the processing efficiency of emotional faces, such as the speed or accuracy when processing faces. Therefore, our review omits studies which focus exclusively on passive viewing of faces, such as looking preferences, electrophysiological recordings, and brain imaging. Additionally, we only examined studies which offer a clear comparison of active behavioral performance for positive emotional faces (i.e., happy faces) against negative emotional faces (e.g., angry, sad, disgusted, afraid faces, etc.). Therefore, a large number of studies which have examined the perception of emotional facial expressions were nevertheless excluded from our overview for reasons such as not offering a direct comparison of positive vs. negative expressions, or only examining passive behavior such as looking-time (e.g., infant studies). Such criteria were applied in order to allow for a straightforward comparison of studies which argue either for positivity or negativity biases in perceiving facial expressions in an analogous manner to our examination of this question for words.

We divided the selected studies on valence effects into two broad categories: Those which used tasks involving the perception of emotional faces on a more basic visual level (detection-based studies with children and adults) which are summarized in Supplementary Table 2A, and those which required the explicit extraction of emotional information from the observed faces presented in Supplementary Table 2B (identification-based studies with children and adults). Examples of the former category include studies which required participants report the position of an odd-one-out face amid non-relevant distractors (the so called “face in the crowd” paradigm), or measured the effectiveness of emotional faces as attentional distractors in visual-perception tasks such as dot-probe detection. These studies address the efficiency with which emotional faces can perceived based upon their visual properties. In contrast to these “detection” tasks, the other category of studies which we examined are “identification” tasks, which require subjects to extract the emotional content of a face, such as in the case of requiring participants to identify the exact emotions displayed by faces. We highlight this distinction between identification and detection based test approaches as it may be a contributor to the differentiation in results supporting either positivity or negativity biases (Leppänen and Hietanen, 2004; Nummenmaa and Calvo, 2015).

### Search Results and Evaluation for Detection-Based Studies

**Figure 2:**
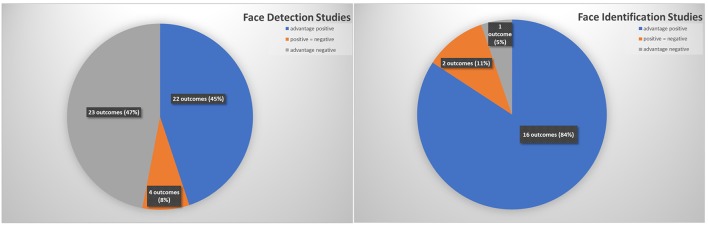
Distribution of outcome measures in face processing studies (absolute numbers based on 22 publications including 49 outcome measures for detection studies and 8 studies including 19 outcome measures for children).

In our overview of detection-type studies, we will consider the 19 studies in Supplementary Table 2A, plus 3 studies from Table 1 (Feyereisen et al., 1986; Schacht and Sommer, 2009; Rellecke et al., 2011) which also include facial expression perception tasks which do not require the identification of particular emotions and can be accomplished through a purely visual assessment of face stimuli. These detection-based studies (22 in total) report 49 outcome results. Figure 2 shows the proportions of findings in favor of positivity (10 studies, 22 outcome results) and negativity biases (11 studies, 23 outcome results), as well as one study which found no significant bias in either direction (Rellecke et al., 2011). Thus, it seems that detection-based studies show a roughly equal tendency of producing results which pointing toward either a positivity or a negativity bias.

The theoretical foundation for the negativity bias stems from ideas about the evolutionary relevance of emotional faces (Baumeister et al., 2001; Vaish et al., 2008): Negative faces such as those displaying anger can serve as a warning of a threat from a rival individual, a fearful face can indicate that a fellow human has sensed an approaching predator, while a disgusted face can convey information regarding the suitability of a plant for consumption. Indeed, 11 of the presented detection studies conclude in favor of a negativity bias with regard to reaction time and in some cases with regard to accuracy as well. That is, negative faces had been processed faster and more correctly than positive faces. However, the other 10 detection studies conclude in favor of the very opposite: A positivity bias, in most cases with regards to reaction time. That is, positive faces have been processed faster. The authors of these studies argue that positive faces (i.e., happy ones) may be more visually distinct compared to negative faces, and are therefore more easily detected by the human visual system (Becker et al., 2011).

In further evaluating detection-based studies, one encounters the inherent difficulty in judging and comparing the visual qualities of the different facial stimuli while comparing studies. The different studies did not only use varying individual stimulus sets, but also qualitatively distinct types of facial stimuli. The most obvious distinction in the types of stimuli used by the detection studies reviewed here is between so called “schematic” faces which provide the most basic concept of a facial emotion (e.g., a circle with two smaller circles for eyes and a mouth curve for a smile) and more detailed facial stimuli such as photographs, computer-generated images, or even detailed drawings which provide a much greater amount of visual detail to the observer. When comparing the detection studies along such a schematic to naturalistic face-type dimension one may notice that all 10 of the studies which conclude in favor of a positivity bias used detailed naturalistic stimuli, while only 4 of the 11 studies which conclude in favor of a negativity bias used such stimuli (Fox and Damjanovic, 2006; Horstmann and Bauland, 2006; LoBue, 2009; Pinkham et al., 2010). The remaining 7 studies which conclude in favor of a negativity bias (White, 1996; Fox et al., 2000; Eastwood et al., 2001, 2003; Ohman et al., 2001; Fenske and Eastwood, 2003; Schlaghecken et al., 2017) all used schematic faces as stimuli. Thus, it seems that schematic faces are more likely to produce results consistent with a negativity bias, while studies using detailed more naturalistic facial stimuli are more likely to produce a positivity bias. It must be investigated in future studies to what extent this conjecture is correct. One possibility could be that negative emotional expressions are simply more pronounced than positive expressions in schematic faces, with the opposite being true in more naturalistic faces, perhaps due to greater difficulty in producing naturalistic posed negative expressions (Leppänen and Hietanen, 2004).

Another explanation for the positivity biases observed by some detection studies is the usage of neutral faces as both targets and distractors in addition to the positive and negative faces. The neutral faces may in fact unbalance the experiment in favor of positive faces, as neutral faces are unusual in everyday life and can be more easily perceived as negative than positive (Leppänen et al., 2004; Tottenham et al., 2013). The usage of these neutral faces may therefore make positive faces appear more distinct by increasing the proportion of faces in the experiment which may be perceived as negative, thereby unbalancing the stimulus selection.

### Search Results and Evaluation for Identification-Based Studies

Regarding identification-based studies, we have examined 6 studies that met our criteria for this review, which are summarized in Supplementary Table 2B plus 2 studies from Table 1. Here we see a much more uniform set of results (see Supplementary Table 2): 84% of the 19 outcome results were in favor of a positivity bias. This could be an indication that while the detection of an emotional face may be highly dependent on the exact testing parameters in terms of whether it shows a positivity or negativity bias, the identification of emotional information from faces might in fact be more efficient for positive faces. However, in the case of the studies in Supplementary Table 2B there is a common confound which cannot be ignored: The positive side of the emotional spectrum contains only a single primary facial expression, happiness, while the negatively-valenced side of the spectrum contains a range of primary facial expressions such as anger, fear, disgust, and sadness, which can be more easily confused for one another (Elfenbein and Ambady, 2003). Therefore, the observed processing advantages for positive faces in the studies in Supplementary Table 2B could also be a result of increased ambiguity in identifying negative facial expressions rather than superior processing of positive facial expressions.

Finally, age may be an important factor in both the detection and identification aspect of perceiving facial emotions. When checking the literature according to the criteria mentioned above we found one detection study and one identification study in which younger and older adults' responses have been compared. The authors observed that younger adults tend to show few differences between the perception of positive and negative faces, while older adults tend to show either improved processing of positive faces (Mather and Carstensen, 2003), or a deficit in the processing of negative faces (Sullivan et al., 2007).

Searching for studies with children we only found a few which met our criteria: 4 detection studies (see Supplementary Table 2A, Walden and Field, 1982; De Sonneville et al., 2002; LoBue, 2009; Zsido et al., 2018) and 2 identification studies (see Supplementary Table 2B, De Sonneville et al., 2002; Tottenham et al., 2013). In all but one of these studies, children's responses (from around 3 to 10 years of age) were compared to adults' responses, and indicated similar results across age. In these studies, all results indicated a positivity bias with regard to reaction time and accuracy, with the exception of the findings of LoBue (2009) which pointed toward a negativity bias. Thus, the majority of studies in which children were included demonstrated a positivity bias across the different task types, such as detection and identification studies. In studies in which only adults were involved, however, the results showed a rather mixed pattern. There were 10 studies (detection-only) which showed a negativity bias, and also 9 studies demonstrating a positivity bias (5 detection and 4 identification studies). The overview of these studies therefore offers some hints that age might in fact play a role in the appearance of positivity and negativity biases. This finding is also supported by the results of a positive/negative face-categorization experiment (Vesker et al., 2018a), where the authors found an initial positivity bias with younger children, but which gradually disappeared, and in some cases even reversed into a negativity bias with increasing age.

## Review 3: Studies on Valence Effects With Words and Faces as Stimuli

By means of the two previous reviews, few studies were identified in the literature search that included both facial and verbal stimuli in their experiments. Since one of our leading questions was if valence effects are specific to the stimulus modality, we conducted a separate literature search using the search term combination “valence AND word AND face.” Criteria for inclusion were as follows:

- Studies must report behavioral results on word AND face processing. If studies used psycho-physiological measures, these studies were also included, but only the behavioral results were extracted for the review.
- In order to compare valence effects across modalities, studies must conduct the same or a similar task with words and with faces in separate experiments. Cross-modal studies that investigate the influence of one modality on the other (priming studies, interference experiments, concurrent presentation of stimuli in both modalities) were excluded.
- Stimuli used in the studies had to be words AND faces differing with respect to valence (positive/negative). Studies were included where positive and negative stimuli were compared directly in a statistical analysis in terms of behavioral results, or where a significant effect was present or absent for one category vs. the other.
- Reported outcomes had to be accuracy and/or reaction times.
- In papers with multiple experiments, the experiments were reported separately whenever they offered a direct comparison of positive vs. negative stimuli.
- Participants had to be healthy children or adults.

After checking ~400 titles and abstracts, four studies could be identified that report valence effects in both modalities along the criteria mentioned above (see Table 1). In these studies, valence effects in adult populations either did not appear at all (Rellecke et al., 2011, accuracy outcomes in Bahn et al., 2017; Vesker et al., 2018a), converged for words and faces (judgement task in Feyereisen et al., 1986), or a valence effect was found in one modality, while no effect of valence emerged in the other modality (categorization task in Feyereisen et al., 1986; Schacht and Sommer, 2009, reaction time outcomes in Bahn et al., 2017; Vesker et al., 2018a).

In the study by Rellecke et al. (2011) participants had to decide whether an emotional stimulus was a word or a face. Here, valence was task-irrelevant. There were no valence effects and no differences between modalities. Schacht and Sommer (2009) also chose a decision task where valence was task-irrelevant: participants had to decide whether a stimulus was a word not, or a face or not, respectively. The results showed no valence effects in lexical decision, but a positivity advantage for face decision. Feyereisen et al. (1986) used two different tasks. In the judgement task (same or different), where valence was task-irrelevant, there was a positivity advantage for words and faces. In contrast, results from the emotional categorization task, where valence was clearly task-relevant, positive words were processed faster than negative words, whereas there was no valence effect for faces. In two parallel studies using a categorization task for audibly presented words (Bahn et al., 2017) and photographs of faces (Vesker et al., 2018a) participants were asked to categorize stimuli as positive or negative as quickly as possible. While the adult groups categorized positive and negative stimuli of both modalities at the same accuracy level, there was a modality effect for reaction times: Adults showed no valence effect for words, but a negativity advantage for faces. The pattern of results becomes even more complex when valence effects in the two modalities are observed across the lifespan. These experiments also included children between 5 and 12 years of age, extending the existing findings on adult processing. Besides converging improvements with age in both modalities (increasing accuracy, decreasing reaction times), the studies revealed age-dependent effects of valence in emotional categorization. A clear early positivity advantage was found in both modalities, most distinct in preschool children. However, the early positivity advantage diminished with age in both modalities and was no longer present or reversed in adulthood. Additionally, the accuracy data of the younger children pointed to another modality effect: The discrepancy between positive and negative items for 6-year olds was stronger for words than for faces, reflecting that negative words were particularly difficult to deal with for young children.

Thus, the overall picture formed by these combined word and face studies (listed in Table 1), is one in which the positivity bias is far more prevalent if any biases are to be found. This might in fact be a direct result of the desire to study words and faces in parallel. Since most studies which would involve the processing of emotion words naturally require participants to process their meaning (at least to some degree), such tasks are conceptually more similar to the identification tasks for faces than detection tasks. Therefore, in attempting to run comparable parallel tasks for both words and faces, it is nearly unavoidable that the most suitable tasks will be ones with a greater tendency of demonstrating positivity biases than negativity biases. This, in turn, leads to the predominant detection of a positivity bias, as described earlier in our review. In fact, the single exception to this tendency was in the study (Vesker et al., 2018a) where participants were deliberately only instructed to classify faces as positive or negative, rather than involving the individual emotions included in the stimulus set, thus avoiding the confounding effect of greater heterogeneity within the negative emotional category for facial expressions. However, even in this study, the negativity effect was found only in adult participants, while all three age groups of children showed a positivity advantage. This divergence of results, even within a single study, therefore serves as a reminder of the crucial influence of development in studying the perception of emotions, as will be further discussed below.

## Discussion and Conclusion

Emotional valence (positive or negative hedonic value) is a crucial and defining feature of emotional stimuli of various kinds. Starting from an obvious heterogeneity in the literature concerning valence effects in the processing of emotional stimuli, we conducted a narrative review of the literature. The aim was to gain a better understanding of the factors that influence the existence and direction of valence effects. We asked whether humans show improved processing of positive or negative stimuli when they perceive words and faces, whether potential valence preferences are modality-specific, and whether valence effects change with age.

With respect to word processing, the literature on adult participants suggest that positive words are processed better than or similarly to negative words, while there was less evidence for a negativity advantage. Data about valence effects for words in children are quite sparse, although the number of studies has increased in recent years. The available findings for children suggest a positivity advantage. For face processing, there are numerous studies with adults, and we focused our review on studies using detection-based and identification-based methods. Detection-based adult studies revealed evidence of both a negativity and a positivity advantage. A final conclusion is difficult to draw as the studies differ with respect to the type of facial stimuli used (schematic or naturalistic), and also regarding the sometimes unbalanced use of positive, neutral, and negative stimuli. Meanwhile, all adult identification studies show evidence of a positivity bias. Children's studies are rarer, but likewise almost exclusively show a positivity bias.

Overall, the present review points to a predominance of findings pointing toward a positivity bias. We will first discuss differences in methodology that might have contributed to the observed pattern before considering potential underlying mechanisms.

First, one must consider the type of task being used. Regarding the influence of task type on word processing, both lexical decision and categorization tasks seem to be associated with a positivity bias (if any valence effects are found). The positivity advantage may be due to the higher informational density of positive verbal stimuli. Alternatively, a negative delay, i.e., the prolonged disengagement of attention from negative stimuli, may have put the negative words at a disadvantage. In contrast, memory tasks seem to promote the processing of negative words. For example, the responses of the adolescents tested by Quas et al. (2016) demonstrated that true and false recognition turned out to be highest for negatively valenced words, followed by the positive and neutral words. The authors assume that this effect arises because negative material is more memorable and malleable, presumably because it is easier to extract the gist from emotionally negative information (Quas et al., 2016, p. 705). A similar suggestion has been put forward by Howe et al. (2010), having shown that false recognition was higher for negative than for neutral words in recognition tasks. Concerning task effects in face processing studies, identification-based, and categorization tasks require participants to process the emotional content of the faces favoring positivity bias results, perhaps due to a greater clustering of positive emotional facial expressions relative to negative ones. By contrast, detection-based tasks which primarily focus on the saliency of target stimuli seem often to produce negativity bias results, perhaps due humans having evolved a greater vigilance to negative stimuli as means of avoid threats.

Second, one must consider differences in the types of stimuli being used. In order to produce a fair comparison of positive vs. negative stimuli it is important to consider fundamental parameters such as arousal and valence across the sets of stimuli being tested, as more extreme stimuli along these parameters will naturally evoke more intense reactions from participants. One must also consider the nature of the stimuli themselves. With respect to words, the semantics of the word stimuli (concrete, abstract, emotional words) might influence the results (see section Evaluation). However, the review suggests that findings of a positivity advantage dominate across different types of words stimuli. Regarding faces, studies using schematic faces (e.g., smilies) tend to show a negativity bias, while those using photographs tend to show a positivity bias, perhaps due to differences in the relative authenticity that can be produced with posed facial expressions (Leppänen and Hietanen, 2004). Another important factor is the overall selection of emotional stimuli used in each experiment. Presenting participants with neutral expressions as part of the experiment may in fact unbalance the procedure as posed neutral faces may be perceived as somewhat negative rather than truly neutral by participants (Tottenham et al., 2013). It might therefore be best to avoid the inclusion of neutral faces entirely in order to create a fair comparison of positive and negative faces.

The third important factor which emerged in this review in terms of determining the presence of categorical emotional biases is the age of participants. Although information about developmental changes in the role of valence in word and face processing during childhood is still sparse, the present review points to some interesting patterns. Experiments on valence effects at different ages point to a positivity advantage in both modalities in children, with differences in accuracy being more pronounced for words than for faces (Bahn et al., 2017; Vesker et al., 2018a). This early bias for positive stimuli confirms the positivity superiority effect observed by Sylvester et al. (2016) for words and findings from Walden and Field (1982), Tottenham et al. (2013), and De Sonneville et al. (2002) for faces. With increasing age, the difference in processing between positive and negative items seems to decrease. In adulthood, the processing of positive words appears to lose its earlier advantage (Bahn et al., 2017), while positive faces even start to show a lower accuracy compared to negative faces (Vesker et al., 2018a).

There are a number of possible explanations for the presence of the early positivity advantage and its subsequent decrease over the course of development into adulthood. First it is possible that younger children simply have less experience with negative emotional stimuli due to spending a lot of time in the protective care of their guardians. This idea is supported by some studies which attempted to quantify the amounts of emotionally positive mother-infant interactions in terms of facial expressions (Ruvolo et al., 2015; Lee et al., 2017). Furthermore, child-directed speech (CDS) serves an important affective role and has often been described as being positively toned. Ponari et al. (2018) suggest that parents are biased toward using positive or avoiding negative language with their children. They support this assumption with a corpus analysis showing that positive words predominated over negative words in caregivers' speech. Of the 50 most frequent words in the corpus, more than half were positive and none were negative. Similarly, Dodds et al. (2015) obtained adults' valence ratings for the 10.000 most frequently used words of 10 languages and found a general positivity bias across languages. Thus, children seem to be exposed to a more positive rather than negative verbal input. This may also explain why positive words are acquired earlier than negative words (Neshat-Doost et al., 1999; Ponari et al., 2018). The earlier age of acquisition may then facilitate positive word processing. However, even when positive and negative word stimuli are carefully controlled for age of acquisition (as in Bahn et al., 2017; Ponari et al., 2018), positive words can still exhibit a processing advantage.

An alternative explanation is that from an evolutionary standpoint prioritizing negative information would not offer children a significant advantage in survival as they would be less able to effectively act on it in order to escape danger compared to adults. In fact, prioritizing positive information may be far more beneficial for children as it would allow them better access to protection from adults and other members of their community. This hypothesis is also supported by findings from studies which examined such effects in older adults, and have found an absence of a negativity bias relative to younger adults. Thus, we believe that biases in perceiving either positive or negative information are dynamic over the lifespan, with children at first showing a positivity bias, which decreases as they grow into younger adulthood. However, the positivity bias seems to re-emerge in elderly participants, possibly due to them experiencing a reduction in their own physical capacities, and perhaps once again beginning to rely more on the protection of others in their community for survival as is the case with children (Mather and Carstensen, 2003; Sullivan et al., 2007, Kappes and Bermeitinger, 2016).

In conclusion, despite our review having a very tight focus, we were able to identify and highlight a number of methodological aspects which can have a significant influence on the outcome of studies examining positivity and negativity biases in the perception of words and facial expressions. Future research should thus carefully consider these aspects (especially task, modality, and stimulus properties), which can influence the appearance, direction, or magnitude of valence effects. In particular, there is a need for developmental studies that might replicate and clarify the early positivity bias.

Finally, the vast majority of the reviewed studies used either words or facial stimuli. Although emotions are typically communicated via language and facial expressions in tandem, fewer studies investigated processing of emotional words and faces with parallel tasks. Results appear to be heterogeneous with respect to modality effects: sometimes the patterns in the two modalities converge, while in other cases there were modality-specific differences. But again, the dominant patterns were a positivity advantage or no valence effect. Beyond the findings from uni-modal studies that were the focus of the present review, mutual influences between the two modalities are another interesting topic. In researching this area, a number of studies have investigated the influence of facial primes on target words and vice versa (e.g., Raccuglia and Phaf, 1997; Aguado et al., 2013; Vesker et al., 2018b). In particular, Raccuglia and Phaf (1997) and Vesker et al. (2018b) converge in finding an asymmetry in cross-modal effects: the effect of facial primes on word processing was smaller than the influence of words on face processing. Future research should explore the interplay of these two modalities in greater detail.

## Author Contributions

All authors listed have made a substantial, direct and intellectual contribution to the work, and approved it for publication.

### Conflict of Interest Statement

The authors declare that the research was conducted in the absence of any commercial or financial relationships that could be construed as a potential conflict of interest.
