# Supplementary material for: The Role of Emotional Valence for the Processing of Facial and Verbal Stimuli—Positivity or Negativity Bias?
Source: Front Psychol. 2019 Jul 26;10:1654. doi: 10.3389/fpsyg.2019.01654 (PMC6676801; doi:10.3389/fpsyg.2019.01654)
Supplement: Supplementary file 1 [file Table_1.pdf]

Supplementary TABLE 1: Summary of studies on valence effects in word processing.

Supplementary TABLE 1A: Summary of studies on valence effects in word processing in adults

ACC: Accuracy, RT: Reaction Times
Adv-pos/neg: behavioral advantage for stimuli with positive / negative valence
pos=neg: no significant difference between positive and negative stimuli.

**Paper number**	**Authors**	**Participants**	**Method/Task**	**Language; Stimuli**	**Results**	**Valence effect Direction**
1	Adelman & Estes (2013)	n=142 under-graduates from Cortese et al. (2010)	Memory task, Recognition memory scores by Corteste et al. (2010) merged with emotionality ratings from Adelman & Estes (2013) Analysis of influence of arousal and valence on memory of words	English; 2578 written positive, negative and neutral words	- Emotion words were remembered better than neutral words<br>- No effect of valence on memory	ACC: Pos = neg
2	Bayer et al. (2012)	n=24 (12 female), mean age = 23.1 years	EEG, Reading, Lexical Decision	German; Written words: 180 nouns taken from BAWL	- Negative words slower than neutral words<br>- Negative words less accurate than positive and neutral words	RT: Adv-pos<br>Acc: Adv-pos
3	Citron et al. (2013)	n=31 (16 female), mean age= 24 years	EEG, Lexical Decision	English; Written words: 150 words, positive - negative - neutral, 150 non-words	- Positive and negative faster than neutral<br>- Positive words more accurate than negative and neutral words<br>- High arousal words more accurate than low arousal words	RT: Pos=neg<br>Acc: Adv-pos
4	Child et al. (2018)	n=36, mean age= 22 years	Reading	English; Written texts: 12positive, 12negative	- No difference in reading time of positive and negative texts	Reading time: Pos =neg
		n=80, mean age= 21 years	Reading + matching/ mismatching condition	English; Written texts: 12positive,<br><br>12negative, 12 with a match of implicit emotional content and explicit emotion word, 12 with mismatch of implicit emotional content and	- No difference in reading time of positive and negative texts	Reading time: Pos =neg

				explicit emotion term		
5	Dijksterhuis & Aarts (2003)	n=25	Word detection (Lexical Decision)	Dutch; Written words: 30 words, positive - negative - neutral	- Negative words detected more often than positive words	Acc: Adv-neg
		n=56	Emotional categorization: Positive vs. negative		- Negative words categorized more accurate than positive words	Acc: Adv-neg
		n=31	Emotional categorization: Positive vs. negative combined with synonymy decision		- Negative word categorized more accurate than positive words<br>- Synonymy decision: positive as accurate as negative	Acc: Adv-neg<br>Acc: Pos = neg
6	Estes & Verges (2008)	n=51	Lexical Decision	English; Written words: 40 words, positive - negative 40 non-words	- Positive words faster than negative words	RT: Adv-pos<br>Acc: Pos = neg
		n=51	Valence Judgement		- Negative faster than positive	RT: Adv-neg<br>Acc: Pos = neg
7	Ferré & Sanchez-Casas (2014)	n=57 (44 female), mean age =19.9 years	Lexical Decision, word-word Priming	Spanish; Written words: 48 concrete words positive-negative	- Positive words faster than negative<br>- Semantic priming effect but no affective priming effect<br>- Participants were faster when words were semantically related	RT: Adv-pos
		n=56 (52 female), mean age =20,3 years		Spanish; Written words: 48 abstract words positive-negative	- Positive words faster than negative<br>- No semantic priming effect<br>- Affective priming: faster reaction times when the valence is congruent	RT: Adv-pos
8	Goh et al. (2016)	n=40	Lexical Decision	English; audibly presented words: 514 concrete nouns 468 non-words	- Positive faster than negative<br>- Faster RT for more concrete words and words with higher number of semantic features<br>- Very negative and very positive faster than neutral<br>- No effect of arousal	RT: Adv-pos

		n=40	Semantic Categorization: abstract vs. concrete		- Positive faster than negative<br>- Faster RT for more concrete words and words with higher number of semantic features<br>- Very negative and very positive faster than neutral<br>- No effect of arousal	RT: Adv-pos
9	Herbert et al. (2006)	n=26 (10 female), mean age = 26 years	EMG and EEG, Valence judgement, Memorization	German; Written words: 180 adjectives, pleasant-unpleasant-neutral	- EMG startle eye blink: blink facilitation for pleasant words	ACC: Adv-pos
10	Herbert et al. (2008)	n= 16 (8 female), mean age = 27 years	EEG, Silent reading; surprise free recall task	German; Written words: 60 pleasant, 60 unpleasant, 60 neutral	- Recall accuracy: better recall for pleasant words than for neutral and unpleasant words	Acc: Adv-pos
11	Hinojosa et al. (2010)	n=32 (28 female), mean age = 23 years	EEG, Word identification (identifying words among nonsense stimuli)	Spanish; Written words: 240 nouns, positive - negative - neutral<br>80 non-recognizable stimuli	- No valence effects	RT: Pos = neg<br>Acc: Pos = neg
			EEG, Word identification (Lexical decision)	Spanish; Written words: 240 nouns, positive - negative - neutral 80 non-words	- Fewer omissions of positive words than of negative and neutral words<br>- Positive words faster than negative and neutral words	RT: Adv-pos<br>Acc: Adv-pos
12	Hofmann et al. (2009)	n=20 (16 female), mean age = 28 years	EEG, Lexical Decision	German; Written words: 200 nouns<br>200 non-words	- Positive faster than neutral<br>- Only negative words with high arousal faster than neutral words<br>- Positive more accurate than neutral<br>- Only negative words with high arousal more accurate than neutral words<br>- Positive and negative words of same arousal: positive words faster and more accurate<br>- Advantage for positive and highly arousing	RT: Adv-pos<br>Acc: Adv-pos

					negative words, arousal not facilitative for positive words	
13	Imbir et al. (2016)	n=32 (15 female), mean age = 21,5	EEG, Lexical Decision	Polish;<br>Written words:<br>135 nouns<br>135 pseudo-words	- Positive words more likely to be classified correctly than negative and neutral words<br>- RT: no effect of valence	Acc: Adv-pos<br>RT: Pos = neg
14	Inaba et al. (2005)	n=16 (10 female), mean age = 22.3 years	EEG, Memorize-recognition task (old vs. new)	Japanese;<br>Written words:<br>150 affective nouns, positive - negative - neutral	- Higher hit rate for positive and negative words than for neutral words<br>- More false alarms for positive than for negative words<br>- Correct responses for positive words faster than correct responses for negative and neutral words	Acc Hit rate: pos = neg<br>Acc False alarms: Adv-neg<br>RT: Adv-pos
15	Itkes & Mashal (2016)	n=40 (23 female), mean age = 25.74 years	Semantic decision task	Hebrew;<br>32 written word pairs: comprising: one head noun and one modifier (positive or negative)	- More correct responses to negative words<br>- Response times to negative word pairs were slower than those of positive word pairs	ACC: Adv-neg<br>RT: Adv-pos
				32 written word pairs: comprising: one head noun and one modifier (positive or negative and either incongruent or congruent with overall valence of the pair)	- No difference between positive and negative word pairs<br>- Responses to negative incongruent word pairs were slower than those to positive incongruent word pairs	ACC: pos=neg<br>RT: Adv-pos
16	Kanske & Kotz (2007)	n=30 (15 female), mean age = 25.6 years	EEG, Lexical Decision	German;<br>Written words:<br>240 nouns, positive-negative-neutral<br>240 non-words	- concrete words faster than abstract words<br>- Positive and negative words faster than neutral words<br>- Concrete positive words faster than concrete negative words	RT: Adv-pos
17	Kappes & Bermeitinger (2016)	Younger adults: n=41 (32 female), mean age = 20.9 years,	Emotional Stroop Task	German;<br>96 written nouns:<br>negative high arousing nouns, negative medium	- Faster responses for positive compared to negative words in younger adults	Younger adults:<br>RT: Adv-pos

		Older adults:<br>n=39 (25 female),<br>mean age = 69.2 years		arousing nouns, positive nouns, neutral nouns	- No differences in response times in older adults<br>- No differences in error rates in both age groups	Older adults:<br>RT: pos=neg<br><br>ACC: pos=neg
18	Kever et al. (2017)	n=60 (8 female),<br>mean age = 20.8 years	Word Decision task (emotional vs non-emotional) in a low- and high-arousing condition	French;<br>66 written emotion related words,<br>32 positive,<br>32 negative,<br>32 neutral	- Positive words were faster recognized than negative and neutral words<br>- Negative words were faster recognized than neutral words	RT: Adv-pos
19	Kissler et al. (2009)	n=20 (10 female),<br>mean age = 9 years	EEG,<br>1. silent reading<br>2. counting of adjectives<br>3. counting of nouns	German;<br>Written words: 198 nouns and adjectives, positive-negative-neutral	- Better memorization of positive words than of negative and neutral words	Acc: Adv-pos
20	Kousta et al. (2009)	n=79 (72 female),<br>mean age = 19,5 years	Lexical Decision	English;<br>Written words: 120 words, positive - negative - neutral<br>120 non-words	- Emotion words faster and more accurate than neutral words<br>- No difference between positive and negative words	RT: Pos = neg<br>Acc: Pos = neg
21	Kuchinke et al. (2005)	n=20 (12 female),<br>20-36 years,<br>mean age = 26.3	fMRI, Lexical Decision	German;<br>Written words: 150 nouns positive - negative - neutral 150 non-words	- Positive faster than neutral and negative words<br>- Positive more accurate than neutral words	RT: Adv-pos<br>Acc: Adv-pos
22	Kuchinke et al. (2007)	n=26 (16 female),<br>18-35 years,<br>mean age = 24.1	Pupillary responses, Lexical Decision	German;<br>Written words: 90 low frequency and 90 high frequency words, positive - negative - neutral<br>180 non-words	High frequency words:<br>- Positive faster than neutral and negative words<br>- accuracy: no difference between negative, positive and neutral<br><br>Low frequency words:<br>- Positive and negative words faster than neutral words<br>- Positive more accurate than negative and neutral words	RT (high freq.): Adv-pos<br>Acc (high freq.): Pos = neg<br><br>RT (low freq.): Pos = neg<br>Acc (low freq.): Adv-pos

23	Kuperman et al. (2014)	Taken from Balota et al. (2007)	Statistical analyses based on existing databases	English; 12658 written words		
			Lexical Decision		- Positive words faster than negative words<br>- Low-arousal words faster than high-arousal words	RT: Adv-pos
			Naming		- Same tendencies but less strong	RT: Adv-pos
24	Lee & Potter (2018)	n=55 (24 female), mean age= 21 years	Facial EMG &cardiac response measured as interbeat interval, Memory task (Forced choice, yes/no)	English; 16 altered radio advertisements with 2 positive, 2 negative,2 neutral target words included	- Positive words were remembered more correctly than neutral words and negative words	ACC: Adv-pos
25	Liu et al. (2016)	n=20 (10 female), mean age= 23,3 years	EEG, Emotional Categorization Task + Memory Task	Chinese Chengyu; 640 written words (traditional Chinese idiomatic expressions): 320 positive 320 negative	- More accurate and faster responses in categorization for positive words compared to negative words<br>- Participants remembered more positive than negative words	Categorization: ACC: Adv-pos RT: Adv-pos<br><br>Recall: ACC Adv-pos
26	Madan et al. (2017)	n=39 (27 female), mean age= 19,9 years	Lexical Decision Free recall task	English; 4 written word lists of 40 words:<br><br>1 with highly arousing taboo words<br>1with positive words<br>1 with negative words<br>1 with neutral words	Lexical decision:<br>- No differences in RTs between positive and negative words<br>- Greater RTs for taboo words compared to RTs for positive and neutral words, but no differences in RTs between taboo words and negative words<br>- Gerater RTs for negative words compared to neutral words<br><br>Recall task:<br>- Taboo words were better recalled than all other word types No differences between other word types	RT (Lexical decision): pos=neg<br><br>ACC (recall): pos=neg
27	Martin & Altarriba (2017)	n=85 (41 female), mean age= 19 years	Lexical decision task with hemifield presentation of words	English; written words: 10 positive emotion words, 10 negative emotion words,	- Positive words faster than negative	RT: Adv-pos

				10 positive emotion-laden words,<br>10 negative emotion-laden words,<br>40 neutral words		
28	Müller & Kuchinke (2016)	n=39 (34 female), mean age= 23.1 years	EEG, Electrooculogram, Lexical Decision	German; Written nouns: 25 happy 25 fear-related 25 neutral<br><br>75 nonwords	- Positive words were faster processed than fear-related and neutral words<br>- No differences in processing speed between neutral and fear-related words<br>- More errors for fear-related words compared to neutral and positive words<br>- No differences between error rates of neutral and positive words	RT: Adv-pos<br><br>ACC: Adv-pos
29	Nasrallah & Carmel (2009)	n=27 (20 female), mean age= 26 years	Emotional categorization: Positive vs. negative	English; Written words: positive - negative - neutral	- Negative words more accurate than positive und neutral words	Acc: Adv-neg
30	Palazova et al. (2011)	n=20 (13 female), mean age= 22.8 years	EEG, Lexical Decision	German; Written words: 180 words, positive - negative - neutral 180 pseudo-words	- Positive words faster than negative words, both faster than neutral words<br>- positive and negative words more accurate than neutral words<br>- high frequent words faster and more accurate than low frequent words<br>- nouns faster and more accurate than adjectives and verbs<br>- words faster and more accurate than pseudo-words	RT: Adv-pos<br>Acc: Pos = neg
31	Palazova et al. (2013)	n=42 (21 female), mean age= 24,1	EEG, Lexical Decision	German; 480 written words: - positive -negative - neutral 480 pseudo-words	- Neutral words faster than positive and negative words<br>- Neutral and negative words more accurate than positive words	RT: Pos = neg<br>Acc: Adv-neg
32	Ponari et al. (2015)	n=95(60 female), mean age= 23,	Lexical Decision	English; Written words: 111 words,	- Overall: positive words faster than negative words,	Overall:<br>RT: Adv-pos

		native English speakers<br><br>n=156 (125 female), English as second language, early vs. late L2		positive - negative - neutral<br>111 non-words	both faster than neutral words<br>- Native + early L2: positive and negative words faster than neutral words, no difference between positive and negative<br>- Late L2: Positive words faster than negative words, both faster than neutral words	Native + Early L2: Pos = neg<br><br>Late L2: Adv-pos
33	Rohr & Abdel Rahman (2018)	n=28 (24 female), mean age 22	EEG, Language production (translation) task + recall task	English; 126 written words, presented in 42 triplets (1 positive, 1 negative, 1 neutral word)	- Participants made more errors in producing the German translation of previously shown English negative words compared to neutral and positive words<br>- No differences in error rates of positive and neutral words	ACC: Adv-pos
34	Santaniello et al. (2018)	n=30 (21 female), age range 20 to 47 years, mean age = 25	EEG, Online recognition memory task	Spanish; 135written nouns:<br>45 positive,<br>45 neutral<br>45 negative	- Faster reactions for negative words compared to positive words<br>- Higher hit rates for negative words compared to positive and neutral words<br>- More false alarms for positive words compared to negative and neutral words	ACC: Adv-neg<br>RT: Adv-neg
35	Scott et al. (2009)	n=26 (15 female), mean age=21	EEG, Lexical Decision	English; 240 written words: - positive<br>-negative<br>-neutral	- Positive and negative words faster than neutral<br>- High frequent faster than low frequent words<br>- Interaction of emotion and frequency<br>- Low frequency: positive and negative words faster than neutral<br>- High frequency: positive faster than negative and neutral	RT: Pos = neg<br>RT: Adv-pos (for high frequent words)
36	Scott et al. (2014)	n=24 (16 female),	Lexical Decision	English; 216 written words, - positive<br>- negative<br>- neutral	- Low frequency: no difference between positive and negative words<br>- High frequency: positive faster	RT: Pos = neg<br>RT: Adv-pos (for high frequent words)

					than negative and neutral	
37	Stenberg et al. (1998)	n=50 (20 female), 20-34 years, mean age (median) = 26	Valence Decision	Swedish; 120 positive and negative words - superimposed on faces (5 angry, 5 happy, 3 neutral expressions)	- Positive faces were categorized faster than negative ones	RT: Adv-pos
38	Võ et al. (2006)	n=21 (13 female), 21-26 years, mean age = 21.45	Valence Decision	German; 60 written affective words: positive, negative neutral	- Positive faster than negative words<br>- Negative words faster than neutral words	RT: Adv-pos
39	Yang et al. (2013)	n=16 (8 female), 21–26 years, mean age = 23.05	EEG, Word counting task (identify identical word stimuli in a row of words)	Chinese; Written words: 24 almost neutral, low and high positive words; 24 almost neutral, low and high negative words	- No effect of valence	RT: Pos = neg
40	Yao et al. (2016)	1. n=19 (10 female), mean age = 21.4 years, native Chinese speakers<br>2. n=20 (9 female), mean age = 22.3 years	EEG, Lexical Decision	Chinese; Written words: 80 concrete 80 abstract 120 pseudo-words	- Concrete words: positive words faster<br>- No difference between high and low arousal<br>- Abstract words: positive words with low arousal faster than high arousal<br>- Negative: high arousal faster than low arousal	RT: Adv-pos
41	Yap & Seow (2013)	n=44	Lexical Decision Replication of Kousta et al. 2009 (see above)	Written words: (Stimuli identical to Kousta et al. 2009)	- Emotion words faster and more accurate than neutral words<br>- No difference between positive and negative words	RT: Pos = neg<br>Acc: Pos = neg
		n=52	go/no go Lexical Decision		- Emotion words faster and more accurate than neutral words<br>- No difference between positive and negative words	RT: Pos = neg<br>Acc: Pos = neg
42	Zhao et al. (2018)	n=18 (9 female), mean age = 20.8 years	EEG, dual-target rapid serial visual presentation task (Memory task)	Chinese; 18 written adjectives: 6 positive 6 negative 6 neutral	- Better presence determination of positive and negative words compared to neutral words<br>- No difference in accuracy between	Acc: Pos = neg

				12 pseudo-words,<br><br>4 strings of four repeated digits	positive and negative words	

Supplementary TABLE 1B: Summary of studies on valence effects in word processing in children

ACC: Accuracy, RT: Reaction Times
Adv-pos/neg: behavioral advantage for stimuli with positive / negative valence
pos=neg: no significant difference between positive and negative stimuli.

**Paper number**	**Authors**	**Participants**	**Method/Task**	**Language; Stimuli**	**Results**	**Valence effect Direction**
1	Bahn et al. (2017)	n in total = 120 5-, 6-, 9-, 12-year-olds and adults,<br>each group: n=24 (12 female)	Lexical Decision	German;<br>48 audibly presented emotion words: 24positive,<br>24 negative<br><br>For lexical decision (additionally):<br>48 audibly presented concrete neutral words<br>96 audibly presented pseudo-words<br><br>All words were recorded with a non-emotional neutral tone	- More correct reactions for positive compared to neutral and negative words in 5-and 6-year-olds<br>- No effect of valence in accuracy in 9- and 12-year-olds and adults<br>- Faster reaction times for negative words in 6-year-olds<br>- No effect of valence in reaction times in all other age groups	ACC:<br>Adv-pos in 5- & 6-year-olds<br>Pos = neg in 9- & 12-year-olds & adults<br><br>RT:<br>Adv-neg in 6-year-olds<br><br>Pos=neg in 5-, 9-, 12-year-olds & adults
			Emotional Categorization		see Table 1 (main text)	see Table 1 (main text)
2	Pérez-Edgar & Fox (2007)	n=65 (29 female),<br>7 years	EEG, Auditory attention task	English;<br>60 audibly presented words repeated 3 times from 5 categories: high positive rating, low social rating; low positive, low social; high positive, high social; low positive, high social; neutral in both	- Positive words faster than negative words	RT: Adv-pos
3	Ponari et al. (2018)	n=60(34 female), 6‑12 years, mean age = 8.09	Lexical Decision	English;<br>24 audibly presented concrete words:<br>7positive	Abstract words:<br><br>- Positive-neutral comparison:<br>- 8- to 9-year-olds recognized	Abstract words:<br>ACC: Pos = neg<br><br>Concrete words:<br>ACC: Pos = neg

		divided in three age groups: 6-7 years; 8-9 years; 10-11 years		7negative<br>7neutral<br><br>24audibly presented abstract words:<br>7positive<br>7negative<br>7neutral	positive words better than neutral words<br>- no differences in 6- to7-year-olds and 10-to 11-year-olds<br><br>- Negative-neutral comparison:<br>- no differences in all age groups<br><br>- Negative-positive comparison:<br>- no differences in all age groups<br><br>concrete words (in all age groups):<br><br>- Positive-Neutral comparison:<br>- Neutral words were better recognized than positive words<br><br>- Negative-Neutral comparison:<br>- Neutral words were better recognized than negative words<br><br>- Positive-Negative comparison:<br>- No differences	
4	Quas et al. (2016)	n=83 (43 female),<br>7 to 8 years, mean age = 7.96, and<br><br>n= 85 (44 female), 12 to 14 years, mean age = 13.46	Memory task	English;<br>180 audibly presented words:<br>60 positive<br>60 negative<br>60 neutral	- Adolescents: Higher number of correctly recalled negative words compared to positive words<br>- Children: No effect of valence on accuracy in recall	ACC Adolescents: Adv-neg<br><br>ACC Children: Pos = neg
5	Salehi et al. (2018)	n=10 (3 female),<br>9 years	EEG, Loud Reading	Farsi<br>120 written words:<br>40 positive<br>40 negative<br>40 neutral	- More correct reactions for positive words, compared to negative words<br>- No differences between positive and neutral words and between neutral and negative words	ACC: Adv-pos<br>RT: Pos = neg
6	Silk et al. (2009)	n=64 (39 female),<br>8.1 to 17.9 years, mean age = 13.2	measure of pupillary reactivity, Valence Decision	English;<br>66 written words<br>positive<br>negative<br>neutral	- No effect of valence	RT: Pos = neg

7	Sylvester et al. (2016)	n=47 (17 female), 9-12 years, mean age = 10.3	Valence Decision	German; 90 written affective words: - positive - negative - neutral	- Positive faster than negative<br>- Negative faster than neutral<br>- Most correct responses for positive words	RT: Adv-pos
8	Vermeulen et al. (2017)	n=386 (212 female), 9–11 years, mean age = 10.5	Memory task	Dutch; Two written word sets of 30 words: 10 positive 10 negative 10 neutral	- Higher number of correctly recalled neutral and positive words, compared to negative words<br>- No differences between positive and neutral words	ACC Adv-pos
9	Zhang et al. (2018)	n=90, 7 to 8 years, mean age = 7.50,<br><br>n=90, 11 to 12 years, mean age = 11.40<br><br>n=90, mean age = 20.37	Memory task	English; 120 audibly presented words: 40 positive 40 negative 40 neutral	- Neutral words were recalled better than negative and positive words<br>- Negative words were recalled better than positive words	ACC Adv-neg

Missing details about the participants' age and gender breakdown (see column 'participants') are due to missing information in the relevant publication.
