# Supplementary material for: The Role of Emotional Valence for the Processing of Facial and Verbal Stimuli—Positivity or Negativity Bias?
Source: Front Psychol. 2019 Jul 26;10:1654. doi: 10.3389/fpsyg.2019.01654 (PMC6676801; doi:10.3389/fpsyg.2019.01654)
Supplement: Supplementary file 2 [file Table_2.pdf]

Supplementary TABLE 2: Studies on face processing in children and adults.

Supplementary TABLE 2A: Detection-based studies on face processing in children and adults.

ACC: Accuracy, RT: Reaction Times
Adv-pos/neg: behavioral advantage for stimuli with positive / negative valence
pos=neg: no significant difference between positive and negative stimuli.

Paper number	Authors	Participants	Method/Task	Stimuli	Results	Valence effect Direction
1	Becker et al. (2011)	Exp. 1A: n=24 adults, (12 female), Exp. 1B: n=25 adults (19 female)	Face in the crowd: Detection of target face among distractor crowds	Angry and happy photographs for targets, neutral photographs for distractors in Exp. 1A, in Exp. 1B only upper halves of faces were used	- Happy faces were detected faster than angry faces, but no differences in accuracy	RT: adv-pos, Acc: pos=neg
		n=30 adults (18 female)		Angry, happy, and neutral computer-generated faces	- Happy faces were detected faster than angry faces, but no differences in accuracy	RT: adv-pos, Acc: pos=neg
		n=72 adults (36 female)	Face in the crowd: Detection of target face among distractor crowds, but with multiple targets present	Angry and happy computer-generated faces with neutral computer-generated faces for distractors	- Happy faces were detected faster and more accurately than angry faces	RT: adv-pos, Acc: adv-pos
		n=21 adults (13 female		Closed-mouth angry and happy photographs with neutral photographs for distractors	- Happy faces were detected faster and more accurately than angry faces	RT: adv-pos, Acc: adv-pos
				Open-mouth angry and happy photographs with neutral photographs for distractors	- Happy faces were detected faster and more accurately than angry faces	RT: adv-pos, Acc: adv-pos
		n=30 adults (20 female)		Closed-mouth angry and happy photographs with afraid photographs for distractors	- Happy faces were detected faster and more accurately than angry faces	RT: adv-pos, Acc: adv-pos
2	De Sonneville et al. (2002)	n=28 7-year-olds (14 female); n=24 8-year-olds (14 female); n=31 9-year-	Yes/No detection task of target emotion, among the other 3	Happy, sad, angry and afraid photographs	- Happy faces detected faster than all other emotion faces in both children and adults<br>- children are also most accurate with happy faces	RT: adv-pos Acc: adv-pos

		olds (16 female); n=23 10-year-olds (10 female); n=26 adults (12 female, mean age 25.2 years)	possible emotions		- ceiling effects flatten accuracy results in adults	
3	Eastwood et al. (2001)	n=11 adults	Detect location of positive or negative face targets in field of neutral face distractors (6, 10, 14, or 18 distractors), face presented in both upright and upside-down conditions	Upright and inverted positive, negative, and neutral schematic faces	- When upright, shallower slope of increasing RT curve with increasing set size for negative faces, and steeper slope for positive faces<br>- Similar slopes when faces are upside-down	RT: adv-neg
4	Eastwood et al. (2003)	n=52 adults	Count number of features in display of schematic faces	Happy and sad schematic faces presented upright and upside-down	- RTs for correct answers longer for negative faces in upright condition, meaning they are stronger distractors. No difference in Inverted condition	RT: adv-neg
		n=28 adults		Happy, neutral, and sad schematic faces presented upright and upside-down	- RTs for correct answers longer for negative faces in upright condition, meaning negative faces are stronger distractors than both happy and neutral faces, and no facilitation from happy faces. No difference in Inverted condition	RT: adv-neg
5	Fenske & Eastwood (2003)	n=40 adults	Flanker interference effect: Detection of target emotional face with same-valence flakers, opposite-valence flankers, neutral-flankers, and no flankers	Happy, sad and neutral schematic faces	- Negative target faces show smaller flanker interference effects and thus hold attention better	RT: adv-neg
6	Fox & Damjanovic (2006)	n=36 adults (22 female)	Face in the crowd: Detection of discrepant stimulus from array of other identical faces	Happy, neutral and angry photographs. Whole faces, just the yes and just the mouth	- Angry faces detected faster with whole faces and eyes-only<br>- but no difference with mouth-only	RT: adv-neg
7	Fox et al. (2000)	n=45 adults (31 female), mean age 22.6 years	Find happy or angry face among neutral face distractors	angry, neutral and happy schematic faces	- Faster RT for detecting Angry faces than happy faces	RT: adv-neg

			(300ms exposure)			
		n=30 adults (19 female), mean age 22.4 years	Face in the crowd: Find happy or angry face among neutral face distractors (800ms exposure)		- Faster RT for detecting Angry faces than happy faces	RT: adv-neg
		n=36 adults (20 female), mean age 24.2 years	Face in the crowd: Find happy or sad face among neutral face distractors in Expression condition, or just the mouth portion (up or down curve among straight line distractors, in Feature condition), 300ms exposure	Expression condition: Sad, neutral, happy schematic faces. Feature condition: Downward curved line, upward curved line, straight line.	- Sad faces detected faster than happy faces in Expression condition, but no differences between upward or downward curved lines in Feature condition.	RT: adv-neg
8	Hodsoll et al. (2011)	n=11 adults, (mean age 27 years, 6 female) in fear condition; n=24 adults (mean age 26 years, 16 female) in happy condition; n=16 adults (mean age 26 years, 9 female) in angry condition	Viewing displays of face tilted either left or right, and having to report the orientation of the target male face while ignoring the 2 distractor female faces. Either the target or one of distractor faces could be emotional, while the other distractor was neutral	Happy, angry, fearful and neutral photographs. Neutral faces were used in all conditions, but only 1 emotion appeared in each of the conditions	- While all emotions slowed RT when used as distractors (compared to neutral distractors), only happy faces also showed facilitation when appearing in the target faces relative to a neutral target face	RT: adv-pos
9	Horstmann & Bauland (2006)	n=20 adults (16 female), mean age 25 years	Face in the crowd: Find target emotional face among 0, 5, or 11 opposite emotion distractors	Happy and angry photographs	- Angry faces detected faster	RT: adv-neg
10	Johnson & Fredrickson (2005)	n=89 Caucasian adults (49 female)	Seen/not-seen recollection task for previously seen joyful, fearful, or neutral faces in same-race (Caucasian) and other-race (African) conditions	Neutral, happy and fearful photographs of Caucasian and African models	- Lowered recognition performance in other-race condition for neutral and fearful faces<br>- but in happy faces performance in the other-race condition was as high as in the same-race condition.	Acc: adv-pos

11	Juth et al. (2005)	n=32 adults (16 female), mean age 26 years	Face in the Crowd: Detection of happy or angry target faces among 7 neutral face distractors	Happy, angry and neutral photographs	- Happy faces detected faster and more accurately than angry faces	RT: adv-pos, Acc: adv-pos
12	LoBue (2009)	n=24 children (5-years old, 12 female), n=24 adults (almost all female), n=12 children and n=12 adults per condition (2 conditions total)	Face in the crowd: Finding target emotion face among opposite emotion distractor (happy versus angry), two conditions, one for each emotion as target with the other acting as the distractor	Happy and angry photographs	- Both children and adults find angry faces faster	RT: adv-neg
			Finding target emotion face among opposite emotion distractor (happy versus afraid), two conditions, one for each emotion as target with the other acting as the distractor	Happy and afraid photographs	- Both children and adults find afraid faces faster	RT: adv-neg
			Finding target emotion face among opposite emotion distractor (happy versus sad), two conditions, one for each emotion as target with the other acting as the distractor	Happy and sad photographs	- Both children and adults find sad faces faster	RT: adv-neg
13	Mather & Carstensen (2003)	n=52 younger adults (30 female, mean age 25.8 years), n=52 older adults (31 female, mean age 74 years)	Dot appears at one of two locations where neutral or emotional faces are previously shown, subjects report location of dot as quickly as possible	60 pairs of photographs, one photo in each pair is always neutral, of the others 20 were happy, 20 sad, and 20 angry	- Older adults: neutral position reported faster than negative position, and positive position reported faster than neutral position<br>- Younger adults: No difference	RT: adv-pos
			Seen/not-seen recollection task for the stimuli viewed in above experiment mixed with novel faces	Same stimuli which were used above with novel distractors	- Older adults: higher accuracy for recalling positive compared to negative faces<br>- Younger adults: No difference	Acc: adv-pos
14	Ohman et al. (2001)	Exp. 1: n=20 adults (8 female), Exp. 2: n=16 adults (6	Face in the crowd: Detection of target face among distractor	Neutral, happy and angry schematic faces	- Overall, angry faces were found faster and more accurately than happy faces	RT: adv-neg, Acc: adv-neg

		female),<br>Exp. 3: n=16 adults (5 female)	crowds of various size			
		Exp. 4: n=36 adults (18 female)	Face in the crowd: Detection of target face among distractor crowds	Vertically inverted neutral, happy, and angry schematic faces	- Overall, angry faces were found faster and more accurately than happy faces	RT: adv-neg,<br>Acc: adv-neg
		Exp. 5: n=18 adults (9 female)		Neutral, happy, "scheming", sad, and angry schematic faces	- Overall, angry faces were found faster and more accurately than all others which were similar to each other	RT: adv-neg,<br>Acc: adv-neg
15	Pinkham et al. (2010)	n=26 adults, mean age 19.46 years	Face in Crowd: Find target emotional face among 8 emotional or neutral distractors	Happy, angry and neutral photographs	- Angry targets were found faster and more accurately than happy targets	RT: adv-neg,<br>Acc: adv-neg
16	Schlaghecken et al. (2017)	n=24 adults, mean age = 21.1 years	Simon task: participants had to respond to the target face by pressing one of two buttons. Target could appear on either right or left side, and so was either congruent or incongruent with the side the correct button was on	Happy and sad schematic faces	- Less interference from target-location/button-location mismatch for negative face targets in terms of both RT and accuracy	RT: adv-neg,<br>Acc: adv-neg
17	Walden & Field (1982)	n=40 children (20 female, mean age 4.5 years),<br>range 3-5 years	Face in the Crowd: Picking face with matching emotion to target faces from array containing all emotions	Drawings (detailed, not simply schematic) of happy, sad, angry and surprised faces	- Highest accuracy with happy faces	Acc: adv-pos
18	White (1996)	n=12 adults in happy condition,<br>n=12 adults in angry condition	Go/No-Go Task: Two grey or blue faces presented on either side of fixation point, subject required to press key as soon as possible if blue color is present in display	Schematic faces. Angry condition: Neutral-Neutral, angry-neutral, neutral-angry face pairs; Happy condition: Neutral-neutral, happy-neutral, neutral-happy pairs	- RT slower in angry condition when angry faces were present<br>- No diff if inverted, or in Happy condition<br>- Thus, angry faces are stronger distractors	RT: adv-neg

19	Zsido et al. (2018)	n=43 pre-school children, mean age=5.65	Detection of a target emotion face from a 3x3 set containing 8 neutral distractor	Children's photographs: 3x3 sets of 8 neutral distractors and 1 target emotion face (fearful, angry, or happy).	- Happy faces detected faster than angry faces<br>- Angry faces detected faster than fearful faces	RT: adv-pos

Supplementary TABLE 2B: Identification-based studies on face processing in children and adults.

ACC: Accuracy, RT: Reaction Times
Adv-pos/neg: behavioral advantage for stimuli with positive / negative valence
pos=neg: no significant difference between positive and negative stimuli.

Paper number	Authors	Participants	Method/Task	Stimuli	Results	Valence effect Direction
1	Calvo & Beltran (2013)	n=25 students (16 female) 18-25 years old	Subjects saw a face presented for a limited duration followed by a word description of an emotion and two-alternative forced choice of whether the word matched the face, ERP data also collected during this task	Happy, angry, sad, fearful and neutral photographs and their word descriptions	- Happy faces trials showed faster RT and accuracy than all other stimuli types	RT: adv-pos<br>Acc: adv-pos
2	De Sonneville et al. (2002)	n=28 7-year-olds (14 female);<br>n=24 8-year-olds (14 female);<br>n=31 9-year-olds (16 female);<br>n=23 10-year-olds (10 female);<br>n=26 adults (12 female, mean age 25.2 years)	Yes/No matching task, where subjects determine if 2 simultaneously presented faces feature the same emotion	Happy, sad, angry and afraid photographs	- Happy faces matched faster and more accurately than all others by both children and adults	RT: adv-pos<br>Acc: adv-pos
3	Kirita & Endo (1995)	n=14 students (7 female)	Subjects were to identify whether sequentially presented faces were happy or sad as quickly as possible	Happy and sad schematic faces presented upright and upside-down	- Happy faces identified faster than sad faces	RT: adv-pos
4	Leppänen & Hietanen (2004)	n=21 adults, mean age 24.7 years	3-choice labeling faces as happy, neutral or disgust	Happy, neutral and disgust photographs	- Happy faces identified faster and more accurately than disgust faces	RT: adv-pos<br>Acc:adv-pos

		n=37 adolescents, mean age 16.3 years	3-choice labeling faces as happy, neutral or sad.	Happy, neutral, and sad schematic faces	- Happy faces identified faster and more accurately than sad faces	RT: adv-pos<br>Acc: adv-pos
5	Sullivan et al. (2007)	n=30 younger adults (15 female, mean age 23 years, range 18-32 years),<br>n=30 older adults (15 female, mean age 72 years, range 60-87 years)	Viewing photos of happy, angry, sad, disgust, fear and surprise and indicating correct label, untimed	Happy, sad, disgust, fear, anger and surprise photographs	- Happy faces identified more accurately than all others<br>- Older adults show accuracy deficits in identifying fear and anger compared to younger adults	Acc: adv-pos
		n=27 younger adults (16 female, mean age 23 years, range 20-37 years),<br>n=27 older adults (14 female, mean age 73 years, range 61-95 years)	Viewing photos of happy, angry, sad, disgust, fear and surprise and indicating correct label, untimed, but with eye-tracking to measure dwell times on faces		- Happy faces identified most accurately<br>- Older adults show deficit in identifying angry faces compared to younger adults	Acc: adv-pos
6	Tottenham et al. (2013)	n=88 children (44 female), 6-17 years old, mean age 12 years	Classifying single faces as pleasant or unpleasant as rapidly as possible	Happy, angry and surprised photographs	- Happy faces categorized faster than angry faces	RT: adv-pos
		n=78 children (43 female), 6-17 years old, mean age 12 years		Happy, angry and neutral photographs	- Happy faces categorized faster than angry faces	RT: adv-pos

Missing details about the participants' age and gender breakdown (see column 'participants') are due to missing information in the relevant publication.
